# Causal association and shared genetics between telomere length and COVID-19 outcomes: New evidence from the latest large-scale summary statistics

**DOI:** 10.1016/j.csbj.2024.05.012

**Published:** 2024-05-10

**Authors:** Jingwei Zhang, Jie Wen, Ziyu Dai, Hao Zhang, Nan Zhang, Ruoyan Lei, Zhixiong Liu, Luo Peng, Quan Cheng

**Affiliations:** a Department of Neurosurgery, Xiangya Hospital, Central South University, Changsha, China; b Hypothalamic Pituitary Research Centre, Xiangya Hospital, Central South University, Changsha, China; c National Clinical Research Center for Geriatric Disorders, Xiangya Hospital, Central South University, Changsha, China; d Department of Neurosurgery, The Second Affiliated Hospital, Chongqing Medical University, Chongqing, China; e College of Life Science and Technology, Huazhong University of Science and Technology, Wuhan, China; f Xiangya School of Public Health, Central South University, Changsha, China; g Department of Oncology, Zhujiang Hospital, Southern Medical University, Guangzhou, China

**Keywords:** Telomere length, COVID-19, Mendelian randomization, Genetic correlation, Mediation analysis

## Abstract

**Background:**

Observational studies suggested that leukocyte telomere length (LTL) is shortened in COVID-19 patients. However, the genetic association and causality remained unknown.

**Methods:**

Based on the genome-wide association of LTL (N = 472,174) and COVID-19 phenotypes (N = 1086,211–2597,856), LDSC and SUPERGNOVA were used to estimate the genetic correlation. Cross-trait GWAS meta-analysis, colocalization, fine-mapping analysis, and transcriptome-wide association study were conducted to explore the shared genetic etiology. Mendelian randomization (MR) was utilized to infer the causality. Upstream and downstream two-step MR was performed to investigate the potential mediating effects.

**Results:**

LDSC identified a significant genetic association between LTL and all COVID-19 phenotypes (rG < 0, *p* < 0.05). Six significant regions were observed for LTL and COVID-19 susceptibility and hospitalization, respectively. Colocalization analysis found rs144204502, rs34517439, and rs56255908 were shared causal variants between LTL and COVID-19 phenotypes. Numerous biological pathways associated with LTL and COVID-19 outcomes were identified, mainly involved in -immune-related pathways. MR showed that longer LTL was significantly associated with a lower risk of COVID-19 severity (OR [95% CI] = 0.81 [0.71–0.92], *p* = 1.24 ×10^-3^) and suggestively associated with lower risks of COVID-19 susceptibility (OR [95% CI] = 0.96 [0.92–1.00], *p* = 3.44 ×10^-2^) and COVID-19 hospitalization (OR [95% CI] = 0.89 [0.80–0.98], *p* = 1.89 ×10^-2^). LTL partially mediated the effects of BMI, smoking, and education on COVID-19 outcomes. Furthermore, six proteins partially mediated the causality of LTL on COVID-19 outcomes, including BNDF, QPCT, FAS, MPO, SFTPB, and APOF.

**Conclusions:**

Our findings suggested that shorter LTL was genetically associated with a higher risk of COVID-19 phenotypes, with shared genetic etiology and potential causality.

## Graphical abstract

**Figure:**
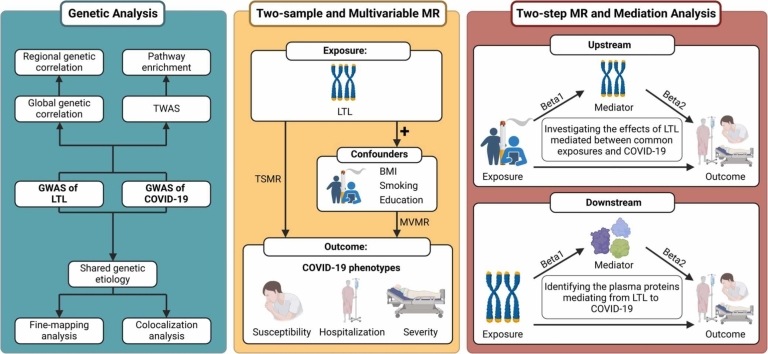
The flow chart of this study explores the causal association and shared genetics between LTL and COVID-19 outcomes. (A) Multiple genetic analyses identify the genetic correlation between LTL and COVID-19 outcomes and potential enriched pathways. (B) TSMR and multivariable MR identify the causal relationship between LTL and COVID-19 outcomes. (C) Two-step MR performs downstream and upstream mediation analyses. Figure created by the biorender.com.

## 1 Introduction

The coronavirus disease 2019 (COVID-19) pandemic caused by severe acute respiratory disease coronavirus 2 (SARS-CoV-2), which emerged in late 2019, is one of the most damaging societal health threats in recent decades and has consistently exerted a subtle impact on human health and socioeconomic conditions. With the gradual exploration of the pathogenesis of COVID-19, there has been a significant decrease in severe COVID-19 cases and associated mortality [1]. Nevertheless, compelling evidence infers that COVID-19 will linger for an extended duration, signifying that multisystem pathological symptoms may persist after infection, now called long COVID [2]. Indeed, it induces long-term pathological alterations in numerous organs, including the circulatory, respiratory, digestive, nervous, and immune systems, manifesting over 200 symptoms, including fatigue, respiratory failure, immune imbalance, and cognitive decline [3]. Currently, the prevalence of long COVID among people is estimated to be 10%, insinuating that at least 76 million individuals worldwide have suffered from long COVID [4]. In particular, long COVID requires increased consideration in older adults (age over 65 or older), as they are more likely to develop persistent symptoms associated with COVID-19 than younger adults. Consequently, an in-depth exploration of the specific role of aging in SARS-CoV-2 infection and exacerbation could assist in effectively alleviating the epidemic of COVID-19 and enhance the effectiveness of rehabilitation in the elderly.

Mammalian telomeres are specialized DNA-protein sequences that promote the formation of a lasso-like structure to maintain chromosomal integrity and regulate the cell cycle during aging [5]. As is well documented, LTL is a key biomarker of biological aging and aging-related pathological changes [6]. Telomere length shortening or structural abnormalities lead to increased incidence and mortality in several age-related chronic diseases, including malignant transformation, dementia, Alzheimer's disease (AD), cardiovascular disease, and anemia [7], [8]. Previous studies have reported that various cumulative exposure factors can regulate telomere length and activity, including educational attainment, smoking status, obesity, alcohol consumption, and physical activity [9], [10]. Interestingly, the relationship between telomere length and COVID-19 risk remains controversial. Several studies have documented that shorter telomere length (LTL) is correlated with higher risks of COVID-19 susceptibility and severity [11]. For instance, Retuerto et al. concluded that the LTL (Z-score) was significantly lower in the COVID-19 hospitalization cohort and was associated with delayed healing of pulmonary abnormalities [12]. Likewise, Diao et al. pointed out that shorter LTL exacerbates SARS-CoV-2-mediated pathological progress by regulating T-cell proliferation and functionally exhausting impairing antiviral immunity in COVID-19 patients [13]. However, recent Mendelian randomization studies have failed to identify substantial evidence supporting a causal correlation between LTL and COVID-19 outcomes [14]. The summary statistics GWAS data used to analyze COVID-19 phenotypes were acquired from an earlier version, and their findings were inconsistent with those of most current observational studies. Therefore, given the availability of recent COVID-19 outcome data, a reanalysis to explore the causality between telomeres and COVID-19 outcomes and the potential mechanism are of paramount importance.

Mendelian randomization (MR), based on detectable single nucleotide polymorphisms (SNPs) as genetic instrumental variables (IVs) for random grouping in large populations, has become a powerful tool for epidemiological investigation studies since it was first proposed by Gray and Wheatley in 1986 [15]. Owing to the random assignment of genetic genotypes, MR can circumvent the confounding effects of bias and reverse causation, thereby enabling an accurate assessment of the causality between exposure and outcome. Superior to traditional observational research methods, MR is less prone to confounders and measurement errors. For example, multivariable Mendelian randomization (MVMR) analysis allows estimation of the causality of multiple exposures to the diseases, regardless of whether the exposure is a confounder, mediator, or collider, which is difficult to perform using traditional observational approaches. Moreover, MR mediation analysis allows unmeasured confounders between exposures, mediators, or outcomes. This paper aimed to explore the genetic correlation, potential signaling pathways, causal relationship, and underlying mediate effect between LTL and COVID-19 outcomes by combining multiple MR and genetic analyses.

## 2 Methods

### 2.1 Study design and GWAS summary data

The flow chart of our research design is displayed in the graphical abstract. All GWAS data were retrieved from publicly available datasets (Supplementary Table 1). There were no imposed restrictions based on gender, age, or income for these GWAS.

GWAS data for the LTL was obtained from the UK Biobank consortium and adjusted for age, sex, and technical factors. A total of 472,174 participants were collected until December 2020 in this GWAS analysis. This study concluded that each LTL 1 SD shorter than the population mean was correlated with a reduction of 2.5 years in life expectancy at the age of 40 years. Genetically determined characteristics of LTL in this GWAS were associated with a broad spectrum of traits, ranging from inflammatory states to aging-related disorders. Moreover, SNPs accounted for 5.64% of the variance in LTL [16].

The GWAS data for COVID-19 were retrieved from the COVID-19-hg GWAS meta-analyses round 7 (https://www.COVID19hg.org/results/r7/), which was the latest version. The GWAS examining COVID-19 susceptibility included 122,616 cases and 2475,240 controls. The GWAS investigating COVID-19 hospitalization included 32,519 cases and 2062,805 controls. Lastly, the GWAS evaluating COVID-19 severity included 13,769 cases and 1072,442 controls [17].

The GWAS data for BMI were gathered from the GIANT consortium, which included 694,649 European participants [18]. The smoking level was quantified by the lifetime smoking index, which was constructed by evaluating both the duration and degree of smoking intensity. The summarized GWAS data on the lifetime smoking index were obtained from the GWAS meta-analysis conducted by Wooton et al., which enrolled 462,690 European participants [19]. Years of schooling represented educational level, and the GWAS data were obtained from the Social Science Genetic Association Consortium (SSGAC), which recruited 766,345 European participants [20]. The proportion of phenotypic variance accounted for BMI, the lifetime smoking index, and educational level were described previously [18], [19], [20].

The GWAS of 4907 plasma protein quantitative trait loci (pQTLs) was obtained from the deCODE genetics dataset Ferkingstad E et al. created, measuring 4907 aptamers in 35,559 Icelanders [21].

Information on the included GWASs is detailed in Supplementary Table 1.

### 2.2 Genetic correlation analyses

Based on the summarized GWAS data, the genetic correlations between the LTL and the COVID-19 risks were determined via linkage disequilibrium score regression (LDSC) [22], [23]. LDSC evaluated the genetic correlation by leveraging the GWAS effect size estimation for a given SNP, which encompasses the effects of all SNPs in linkage disequilibrium (LD) with that SNP. First, all SNPs were harmonized using munge_sumstats.py. Then, the genetic correlations were estimated utilizing the ldsc.py with pre-computed LD scores of 1000 Genome European data [24]. *P* < 0.05 was considered a significant correlation.

The SUPERGNOVA was used to calculate the pairwise local genetic correlation [25]. The algorithm partitions the entire genome into roughly 2252 linkage disequilibrium-independent blocks, precisely measuring the similarity between paired traits influenced by genetic variations within each block. Statistical significance was established using Bonferroni-adjusted *p*-value (*p* < 0.05/2252), and 0.05/2252 < *p*-value < 0.05 was considered as suggestive [26].

### 2.3 Multi-trait analysis of GWAS (MTAG)

To ascertain risk SNPs linked to the composite phenotype comprising LTL and COVID-19 outcomes, we conducted a cross-trait meta-analysis using MTAG with GWAS summary statistics [27]. The MTAG approach presumes equal SNP heritabilities across traits and consistent genetic covariances between them. This method accommodates potential sample overlap among GWAS, as previously described [28]. To select independent SNPs, we implemented PLINK clumping (r^2^ = 0.05, distance = 1000 kb). Then, the independent shared SNPs not driven by a single trait (5 ×10^-8^ < *p*_*single-trait*_ < 5 ×10^-3^) were considered novel shared SNPs.

### 2.4 Colocalization analysis

We conducted a colocalization assessment employing the Coloc tool [29]. Summary statistics were compiled for variants within 500 kb of the index SNP at every shared locus. We then computed the posterior probabilities for H4 (PP.H4, indicating that both traits are associated via a single causal variant) and H3 (PP.H3, reflecting the likelihood that each trait is linked to different causal variants). A locus was deemed colocalized if the PP.H4 exceeded 0.5.

### 2.5 Fine-mapping credible set analysis

We further identified a 99% credible set of causal variants using an efficient Bayesian fine-mapping method called FM-summary, detailed in the FM-summary GitHub Repository [30]. For this process, we gathered variants within a 500 kb radius of each significant index SNP identified from cross-trait meta-analysis to input into the FM summary. This method estimated each variant's posterior inclusion probability (PIP), indicating the probability of a true association with the trait or disease. To establish the 99% credible set, we sorted the SNPs in order of decreasing PIP and summed these probabilities until the sum was at least 99%. More detailed explanations are provided in previous descriptions [31], [32].

### 2.6 Transcriptome-wide association study (TWAS) and enriched signaling pathways analysis

As described in a previous article [33], GWAS were converted to TWAS by the FUSION method, which used a linear model of expression quantitative trait loci (eQTL) to predict gene expression based on an RNA-seq reference plate. This study used European Lung RNA-seq Genotype-Tissue Expression version 8 (GTEx v8) as reference plates [34]. Homozygous gene clusters significantly associated with LTL and COVID-19 outcomes were identified through the TWAS method. To further elucidate the underlying biological mechanisms common to LTL and COVID-19 outcomes, biological pathway enrichment analysis of these genes was performed based on the Gene Ontology database using R packages (clusterProfiler [35], enrichplot [36], and DOSE [37]). *P* for LTL and COVID-19 outcomes were combined using Fisher's Combined P-value (FCP) method. Protein-protein interaction was plotted by GeneMANIA [38].

### 2.7 Instrumental variable (IV) acquisition

To ensure the reliability of the genetic instruments selected for MR analysis, the genome-wide correlation *p*-value threshold was set to 5 × 10^-8^. Then, based on the 1000 Genomes Project of the European samples, linkage disequilibrium analysis (r^2^ < 0.001) was performed to further screen for independent IVs. As previously described, the F statistic for each IV in the exposure was calculated using the following formula: (R^2^/K)/[(1-R^2^) (N-K-1)], where K denotes the number of SNPs and N represents the sample size [39], [40]. R^2^ is the variance explained by SNPs, calculated following the formula: 2 *EAF* (1-EAF)* (Beta/SE)^2^. Only IVs with F > 10 were retained to guarantee robust instruments. After extracting the IVs, we used the harmonise_data function from the TwoSampleMR package [41] to perform the harmonization under default settings as previously described (https://mrcieu.github.io/TwoSampleMR/articles/harmonise.html).

### 2.8 Two-sample Mendelian randomization (TSMR)

Three methods were utilized for TSMR analysis: random-effects inverse variance weighting (IVW), weighted median, and MR Egger, with IVW serving as the primary method and the latter two providing supplementary analyses. IVW employs a weighted regression that maps IV outcome effects onto exposure effects, assuming a fixed intercept of zero, which offers the highest statistical power. However, horizontal pleiotropy in IVs can confound results through pathways unrelated to the exposure. Thus, the weighted median and MR Egger methods were also employed, as they are more resistant to the effects of horizontal pleiotropy despite a potential reduction in statistical power [42]. The weighted median method utilizes the median of the MR estimates for causal inference [43]. In contrast, MR Egger regression does not fix the intercept, allowing it to be freely estimated to account for the average effect of pleiotropy across the IVs [44]. Concerning dichotomous outcomes, effects were quantified as beta (log (OR)).

Two-sample Mendelian randomization utilized (GWAS) summary statistics to infer the presence and strength of causal effects between exposure and outcome. However, the overlap between the exposure and outcome samples may introduce bias and thus affect the reliability of the conclusions [45], [46]. Ninon Mounier et al. [47] developed a method (MRlap) that simultaneously considers weak instrument bias and winner's curse while modeling the entire instrument selection process, sample overlap, and exposure effect estimation error. This approach minimizes bias in estimating causal effects and facilitates the inclusion of substantially larger GWAS sample sizes by accommodating potentially overlapping samples.

To assess the robustness of our findings, we analyzed horizontal pleiotropy using leave-one-out tests and the MR Egger intercept test to check for deviations from the null hypothesis [48]. These tests vary in their basic assumptions but are primarily used to evaluate the extent to which the influence of one or more instrumental SNPs is exaggerated. This overestimation may occur not only through the proposed causal pathway but also through other unrecognized causal pathways.

In Mendelian randomization analyses, surpassing an efficacy benchmark of 80% is frequently advised [49], [50]. Attaining this efficacy standard confirms that the study possesses ample robustness to identify an authentic causal relationship between the genetic variants employed as instrumental variables and the targeted outcome. A power level exceeding 80% diminishes the likelihood of Type II errors (the oversight of a genuine effect), thus bolstering the dependability of the study outcomes. MR power was calculated using an available web tool (https://sb452.shinyapps.io/power).

### 2.9 Multivariable Mendelian randomization (MVMR)

MVMR allows the estimation of causality between multiple exposures and a single outcome, regardless of whether the exposure is a confounder, mediator, or collider[51]. It is also suitable for analyzing variables with pleiotropy and acts as another form of sensitivity analysis. MVMR allows researchers to examine the effects of multiple potential mediating variables on the outcome variable simultaneously, taking into account more genetic variation and thus making more comprehensive use of the available information and improving statistical validity. Herein, MVMR was performed to estimate the direct effects of LTL on COVID-19 phenotypes, adjusting for BMI, smoking, and education. As the number of confounders included in MVMR increased, the power of the analysis decreased [52]. Thus, our MVMR model only introduced one potential confounder for each analysis.

### 2.10 Two-step Mendelian randomization for mediation analysis

Many phenotypes affecting LTL are also risk factors for COVID-19 outcomes; however, whether LTL plays a vital role in mediating these risk factors and COVID-19 remains elusive. Meanwhile, the mediating mechanism by which LTL leads to COVID-19 outcomes remains unclear. Thus, a mediation analysis was carried out to investigate these potential mediated effects. Although MVMR could identify the mediated effects, estimating the combined effects of multiple mediators is more appropriate. Herein, a two-step MR approach was chosen to conduct mediation analysis to calculate the individual mediating effect of LTL owing to its relatively low risk of bias [52]. BMI, Smoking, and education level were selected as the common risk factors for telomere length and COVID-19 based on the observation of previous studies [53]. Moreover, a two-step MR approach was used to explore the mediating role of plasma proteins between LTL and COVID-19 outcomes. Genetic IVs for these mediators were chosen based on *p*-values of 1 × 10^-5^ as previously described [54]. For dichotomous variants, OR was converted into log OR (beta) to quantify the mediated effects using the coefficient product method. The proportion of the mediated effect was quantified as the mediated effect divided by the total effect, and the standard error (SE) was computed by the delta method.

### 2.11 Statistical analysis

MR results were illustrated as OR, beta, SE, and 95% confidence interval (95% CI). To address multiple testing in the TSMR, a Bonferroni-corrected *p*-value 0.00833 (0.05/1 *3 *2) was considered significant, with a 0.05 < *p*-value < 0.0033 being regarded as suggestive. The R software package (Two-Sample MR, version 0.5.6) was utilized for statistical analyses.

## 3 Results

### 3.1 Overview

Multiple genetic analyses identified the shared genetic etiology between LTL and COVID-19 outcomes. Meanwhile, many hub gene transcripts and significantly enriched biological pathways associated with both LTL and COVID-19 outcomes were explored through TWAS (Graphical Abstract (A)). In addition, the results of TSMR and MVMR demonstrated the significant causality between LTL and COVID-19 outcomes after adjusting for several confounders (BMI, smoking, and educational level) (Graphical Abstract (B)). Furthermore, our results exposed that LTL partly mediated the causality between common risk factors (BMI, smoking, and educational level) and COVID-19 outcomes. We also calculated the mediating effect of plasma proteins between LTL and COVID-19 outcomes (Graphical Abstract (C)).

### 3.2 Genetic correlation analysis between LTL and COVID-19 outcomes

**Fig. 1:**
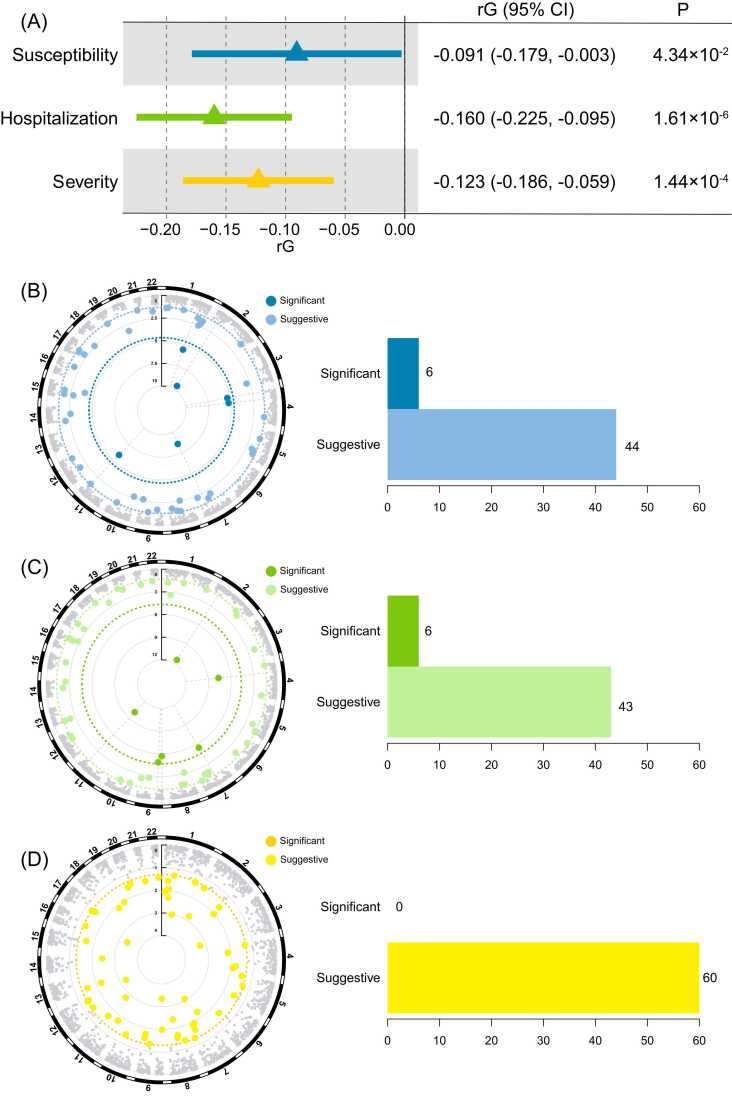
Genetic correlation analyses between LTL and COVID-19 outcomes. (A) LDSC calculates the genetic correlation between LTL and COVID-19 outcomes. SUPERGNOVA calculates the genetic correlation between LTL and COVID-19 susceptibility (B), hospitalization (C), and severity (D). Significant represents a *p-*value less than 0.05/2252; Suggestive represents a *p-*value between 0.05/2252 and 0.05. Blue color represents COVID-19 susceptibility; Green color represents COVID-19 hospitalization; Yellow color represents COVID-19 severity; 95%CI: 95% confidence interval; rG: genetic correlation.

Firstly, a significant genetic relationship was identified between LTL and COVID-19 outcomes using LDSC analysis (Fig. 1A). Briefly, genetic correlation analyses identified a significant negative genetic correlation between LTL and COVID-19 susceptibility (rG [95% CI] = -0.09 [-0.18, -0.003], *p* = 4.34 ×10^-2^), hospitalization (rG [95% CI] = -0.16 [-0.23, -0.10], *p* = 1.61 ×10^-6^), and severity (rG [95% CI] = -0.12 [-0.19, -0.06], *p* = 1.44 ×10^-4^). Partitioning the whole genome into LD-independent regions, six significant and 44 suggestive regions were found for COVID-19 susceptibility (Fig. 1B); six significant and 43 suggestive regions were found for COVID-19 hospitalization (Fig. 1C); 60 suggestive regions were found for COVID-19 severity (Fig. 1D). Details are shown in the Supplementary Table 2.

### 3.3 Identiﬁcation of shared genetic etiology and colocalization between LTL and COVID-19 outcomes

**Fig. 2:**
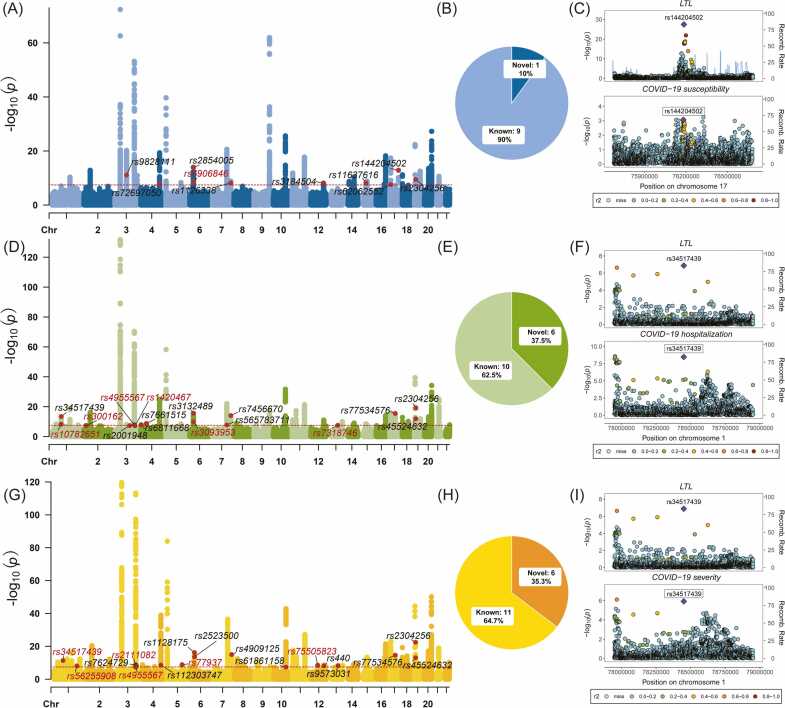
Shared variants between LTL and COVID-19 phenotypes by cross-traits GWAS meta-analysis. Manhattan plot of MTAG between LTL and COVID-19 susceptibility (A), hospitalization (D), and severity (G). The pie plot shows the number of novel and known shared SNPs between LTL and COVID-19 susceptibility (B), hospitalization (E), and severity (H). The most significant shared SNPs between LTL and COVID-19 susceptibility (C), hospitalization (F), and severity (I). Red color represents novel shared SNPs, and black color represents known associated SNPs.

Based on the significant genetic correlations identified between leukocyte telomere length (LTL) and COVID-19 susceptibility, hospitalization, and severity, we conducted cross-trait GWAS to find shared risk SNPs associated with LTL and COVID-19 outcomes using the MTAG method (Fig. 2 and Supplementary Table 3). One novel SNP and nine known SNPs were found to associate with LTL and COVID-19 susceptibility (Figs. 2A and 2B). Among these 10 shared SNPs, rs144204502 was found to colocalize between LTL and COVID-19 susceptibility (PP.H4 = 0.66) (Fig. 2C and Supplementary Table 4), which is located near *TK1* and encodes a cytosolic enzyme that catalyzes the addition of a gamma-phosphate group to thymidine [55]. Six novel SNPs and ten known SNPs were found to be associated with LTL and COVID-19 hospitalization (Figs. 2D and 2E). Among them, rs34517439 was colocalized between these two traits (PP.H4 = 0.99) (Fig. 2F and Supplementary Table 4), which is located near *DNAJB4* and encodes a protein that binds to the cell adhesion protein E-cadherin and targets it to the plasma membrane [56]. Six novel SNPs and 11 known SNPs were found to be associated with LTL and COVID-19 severity (Figs. 2G and 2H). Among them, rs34517439 (PP.H4 = 0.99) and rs56255908 (PP.H4 = 0.71) were the colocalized variants between LTL and COVID-19 severity (Fig. 2I and Supplementary Table 4). rs56255908, located near *CAMSAP2,* has microtubule minus-end binding activity [57]. Interestingly, rs34517439 was colocalized in both trait pairs of LTL & COVID-19 hospitalization and LTL & COVID-19 severity. For each MTAG-identified significant locus, we identified a 99% credible set of causal SNPs employing FM-summary for further determination; details were shown in Supplementary Table 5.

### 3.4 Hub gene and pathway enrichment analyses

Then, TWAS was conducted to identify hub downstream genes and biological signaling pathways associated with LTL and COVID-19 outcomes (Supplementary Tables 6–8). A total of 20 hub genes associated with LTL and all COVID-19 outcomes in the reverse direction were identified via the TWAS analysis (Supplementary Table 9 and Supplementary Figure 1A). Protein-protein interaction was constructed for these 20 hub genes (Supplementary Figure 1B). The top 10 hub genes were *CCHCR1, CCR5, HLA-C, PMF1, LRRC37A, CASTOR3, IFNAR1, SLC25A44, DR1*, and *MACF1*. The significant enriched biological pathways associated with LTL and COVID-19 susceptibility are detailed in Supplementary Table 10 and Supplementary Figure 2 A, including protein transmembrane transport, cellular aldehyde metabolic process, and mitotic G1 DNA damage checkpoint signaling. The significant enriched biological pathways associated with LTL and COVID-19 hospitalization are detailed in Supplementary Tables 11 and Supplementary Figure 2B, including movement in the host environment, the biological process involved in interaction with the host, and viral entry into the host cell. The significant enriched biological pathways associated with LTL and COVID-19 severity are detailed in Supplementary Tables 12 and Supplementary Figure 2C, including antigen processing and presentation of peptide antigen, regulation of phosphatase activity, and protein targeting to lysosome.

### 3.5 Longer LTL was causally associated with lower risks of adverse COVID-19 outcomes

**Fig. 3:**
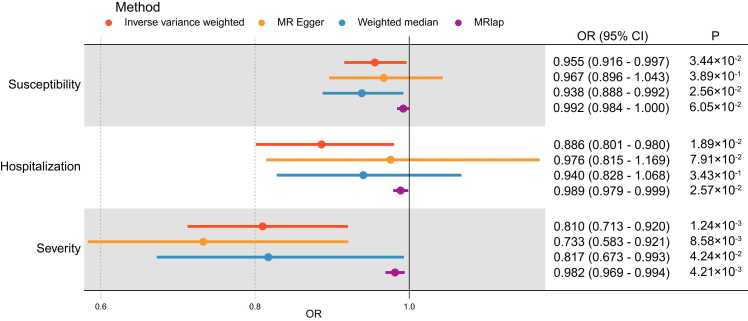
IVW, MR Egger, weighted median, and MRlap results of the causal relationship between LTL and COVID-19 outcomes. Red color represents the Inverse variance weighted method; Yellow color represents the MR Egger method; Blue color represents the Weighted median method; Purple color represents the MRlap method; 95%CI: 95% confidence interval; OR: odds ratio; OR [95% CI] per 1 SD increase in predicted LTL.

Afterward, IVW, MR Egger, weighted median, and MRlap were used to calculate the causal effect of LTL on COVID-19 outcomes (Fig. 3). The selection of IVs for LTL and COVID-19 outcomes was detailed in Supplementary Tables 13–16. The F value for the IVs ranged from 28.7 to 1542.9, showing robust reliability. IVW analysis indicated that longer LTL was significantly correlated with a lower risk of COVID-19 severity (OR [95% CI] = 0.81 [0.71, 0.92], *p* = 1.24 ×10^-3^). Longer LTL was suggestively in relation to lower risks of COVID-19 susceptibility (OR [95% CI] = 0.96 [0.92, 1.00], *p* = 3.44 ×10^-2^) and COVID-19 hospitalization (OR [95% CI] = 0.89 [0.80, 0.98], *p* = 1.89 ×10^-2^)(Supplementary Table 17). Moreover, to reduce bias due to the partial overlap of exposure and outcome samples, we validated our conclusions using MRlap. The results of MRlap showed that longer LTL was significantly correlated with a lower risk of COVID-19 severity (OR [95% CI] = 0.98 [0.97, 0.99], *p* = 4.21 ×10^-3^) and was suggestively correlated with a lower risk of COVID-19 hospitalization (OR [95% CI] = 0.99 [0.98, 1.00], *p* = 2.57 ×10^-2^). The power for the MR correlation of LTL with COVID-19 susceptibility, hospitalization, and severity was 85.7%, 98.6%, and 99.7%, respectively (Supplementary Table 18), showing strong robustness.

Conversely, our analysis did not reveal significant causalities of COVID-19 outcomes, including susceptibility (*p* = 0.42), hospitalization (*p* = 0.49), or severity (*p* = 0.90), on LTL (Supplementary Table 17).

The pleiotropic analysis showed the absence of pleiotropy (Supplementary Table 19). Leave-one-out analysis did not reveal unusual SNPs (Supplementary Figure 3).

### 3.6 The causal effects between LTL and COVID-19 remain significant after adjusting for BMI, smoking, and educational level

**Fig. 4:**
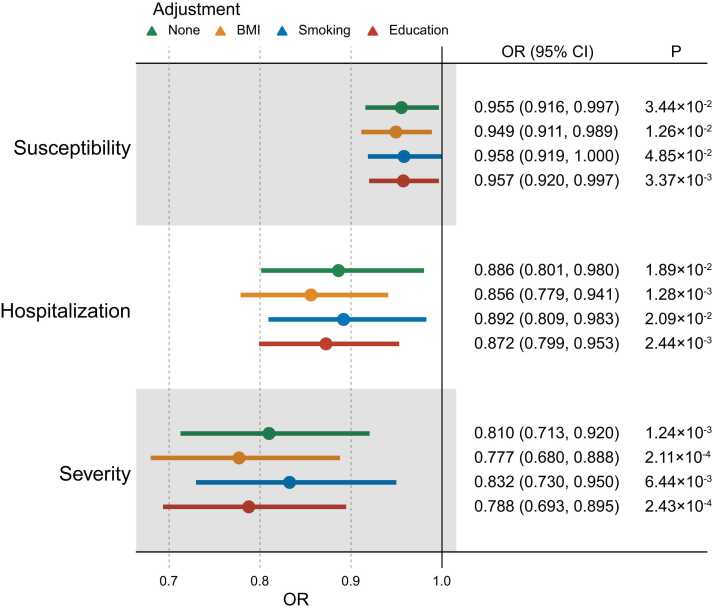
MVMR results of the causal relationship between LTL and COVID-19 outcomes adjusting for BMI, smoking, and education. Green color represents no adjustment; Yellow color represents an adjustment to BMI; Blue color represents an adjustment to smoking; Dark red color represents an adjustment to education; 95%CI: 95% confidence interval; OR: odds ratio; BMI: body mass index; OR [95% CI] per 1 SD increase in predicted LTL.

MVMR was utilized to calculate the direct effects of LTL on COVID-19 outcomes after adjusting for BMI, smoking, and educational level (Fig. 4). IVW analysis suggested that longer LTL was correlated with lower COVID-19 susceptibility (OR [95% CI] per 1 SD increase in predicted LTL = 0.95 [0.91, 0.99], *p* = 1.26 ×10^-2^), hospitalization (OR [95% CI] = 0.86 [0.78, 0.94], *p* = 1.28 ×10^-3^), and severity (OR [95% CI] = 0.78 [0.68, 0.89], *p* = 2.11 ×10^-4^) after adjusting for BMI. Moreover, the analysis determined that longer LTL was related to lower COVID-19 susceptibility (OR [95% CI] = 0.96 [0.92, 1.00], *p* = 4.85 ×10^-2^), hospitalization (OR [95% CI] = 0.89 [0.81, 0.98], *p* = 2.09 ×10^-2^), and severity (OR [95% CI] = 0.83 [0.73, 0.95], *p* = 6.44 ×10^-3^) after adjusted for smoking level. Of note, longer LTL was related to lower COVID-19 susceptibility (OR [95% CI] = 0.96 [0.92, 1.00], *p* = 3.37 ×10^-3^), hospitalization (OR [95% CI] = 0.87 [0.80, 0.95], *p* = 2.44 ×10^-3^), and severity (OR [95% CI] = 0.79 [0.69, 0.90], *p* = 2.43 ×10^-4^) after adjusted for educational level. Overall, the causal effects between LTL and COVID-19 were all significant after adjusting for potential mediators, including BMI, smoking, and educational level, indicating the robustness of our findings.

### 3.7 LTL acted as a mediator between several risk factors and COVID-19 outcomes

**Fig. 5:**
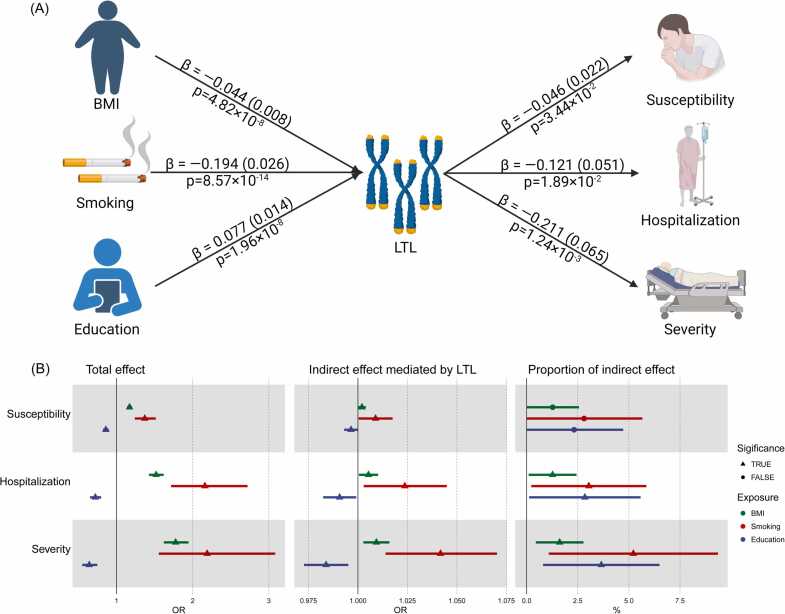
Identify the mediating role of LTL between BMI, smoking, education, and COVID-19 outcomes. (A) Beta (log (OR)) and *p* values for the effect of LTL between BMI, smoking, education, and COVID-19 outcomes. (B) Forest plot demonstrating the potential mediating role of LTL between BMI, smoking, education, and COVID-19 outcomes. The black triangle represents statistical significance; The black circle represents no statistical significance; The green color represents BMI; Red color represents smoking; The dark blue color represents education; OR: odds ratio; BMI: body mass index; OR [95% CI] per 1 SD increase in predicted LTL.

Next, a two-step MR approach was employed to investigate the potential mediating role of LTL between BMI, smoking, educational level, and COVID-19 outcomes (Fig. 5 and Table 1). IVW analysis indicated that BMI (beta = -0.04, *p* = 4.82 ×10^-8^) and smoking level (beta = -0.19, *p* = 8.57 ×10^-14^) were negatively associated with LTL (Fig. 5A and Supplementary Table 20). Moreover, educational level (beta = 0.08, *p* = 1.96 ×10^-8^) was found to be positively associated with LTL (Fig. 5A and Supplementary Table 20). The total effect and indirect effects of BMI, smoking level, and educational level on COVID-19 outcomes are presented in Fig. 5B, Table 1, and Supplementary Table 20. In short, the proportion of indirect effect mediated by LTL between BMI and COVID-19 susceptibility, hospitalization, and severity were 1.27% (95% CI: 0–2.56%, *p* = 4.86 ×10^-2^), 1.27% (95% CI: 0.10–2.44%, *p* = 3.11 ×10^-2^), and 1.61% (95% CI: 0.45–2.78%, *p* = 5.46 ×10^-3^), respectively. At the same time, the proportion of indirect effect mediated by LTL between smoking level and COVID-19 susceptibility, hospitalization, and severity was 2.81% (95% CI: 0–5.66%, *p* = 4.19 ×10^-2^), 3.04% (95% CI: 0.23–5.85%, *p* = 2.51 ×10^-2^), and 5.22% (95% CI: 1.08–9.36%, *p* = 3.05 ×10^-3^), respectively. The proportion of indirect effect mediated by LTL between educational level and COVID-19 susceptibility, hospitalization, and severity was 2.32% (95% CI: 0–4.72%, *p* = 4.78 ×10^-2^), 2.85% (95% CI: 0.13–5.57%, *p* = 3.03 ×10^-2^), and 3.65% (95% CI: 0.80–6.50%, *p* = 5.13 ×10^-3^), respectively.

Figure created by the biorender.com.

**Table 1:** Causal effects of BMI / Smoking / Education on COVID-19 mediated by LTL.

	BMI			Smoking			Education		
	Total effect (95% CI)	Indirect effect (95% CI)	Proportion (%) (95% CI)	Total effect (95% CI)	Indirect effect (95% CI)	Proportion (%) (95% CI)	Total effect (95% CI)	Indirect effect (95% CI)	Proportion (%) (95% CI)
Susceptibility	1.171 (1.138, 1.205) P = 2.91 × 10^-27^	1.002 (1.000, 1.004) P = 4.86 × 10^-2^	1.273 (0, 2.559)	1.370 (1.240, 1.515) P = 6.69 × 10^-10^	1.009 (1.000, 1.018) P = 4.19 × 10^-2^	2.809 (0, 5.657)	0.859 (0.821, 0.899) P = 6.81 × 10^-11^	0.996 (0.993, 1.000) P = 4.78 × 10^-2^	2.320 (0, 4.722)
Hospitalization	1.519 (1.426, 1.619) P = 4.59 × 10^-38^	1.005 (1.000, 1.010) P = 3.11 × 10^-2^	1.268 (0.099, 2.437)	2.160 (1.716, 2.720) P = 5.70 × 10^-11^	1.024 (1.003, 1.045) P = 2.51 × 10^-2^	3.040 (0.228, 5.853)	0.721 (0.652, 0.798) P = 2.16 × 10^-10^	0.991 (0.982, 0.999) P = 3.03 × 10^-2^	2.849 (0.125, 5.573)
Severity	1.775 (1.620, 1.945) P = 7.41 × 10^-35^	1.009 (1.003, 1.016) P = 5.46 × 10^-3^	1.614 (0.447, 2.781)	2.189 (1.554, 3.084) P = 7.41 × 10^-6^	1.042 (1.014, 1.070) P = 3.05 × 10^-3^	5.219 (1.080, 9.358)	0.641 (0.550, 0.747) P = 1.27 × 10^-8^	0.984 (0.973, 0.995) P = 5.13 × 10^-3^	3.650 (0.802, 6.498)

### 3.8 Plasma proteins acted as mediators between LTL and COVID-19 outcomes

**Fig. 6:**
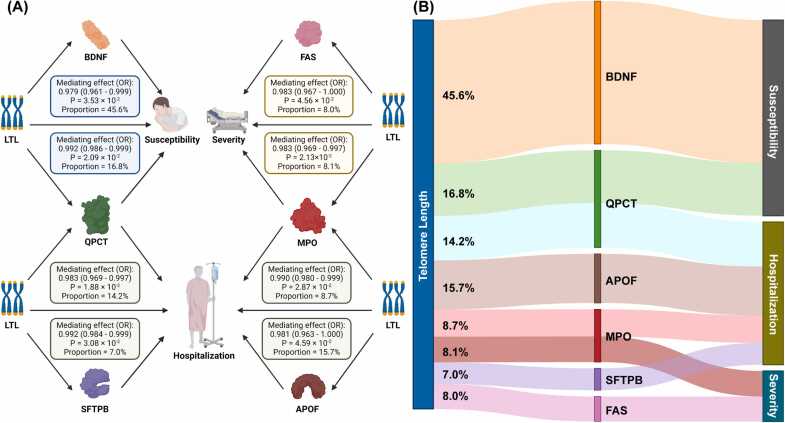
Identify the mediating role of plasma protein between LTL and COVID-19 outcomes. (A) Mediating effect and proportion of plasma protein between LTL and COVID-19 outcomes. (B) Mediators from LTL to COVID-19 outcomes identified in two-step MR. The width of each strip indicates the mediating proportion; BDNF: plasma brain-derived neurotrophic factor; QPCT: glutaminyl-peptide cyclotransferase; APOF: apolipoprotein F; MPO: myeloperoxidase; SFTPB: surfactant protein B; OR: odds ratio.

Finally, we investigated the potential mediating role of plasma proteins between LTL and COVID-19 outcomes (Fig. 6 and Table Supplementary Table 21). In summary, we found that BDNF (OR [95% CI] = 0.98 [0.96, 1.00], *p* = 3.53 ×10^-2^, proportion = 45.6%) and QPCT (OR [95% CI] = 0.99 [0.99, 1.00], *p* = 2.09 ×10^-2^, proportion = 16.8%) partially mediated the causal effect between LTL and COVID-19 susceptibility. QPCT (OR [95% CI] = 0.98 [0.97, 1.00], *p* = 1.88 ×10^-2^, proportion = 14.2%) and SFTPB (OR [95% CI] = 0.99 [0.99, 1.00], *p* = 3.08 ×10^-2^, proportion = 7.0%) partially mediated the causal effect between LTL and COVID-19 hospitalization. MPO (OR [95% CI] = 0.99 [0.98, 1.00], *p* = 2.87 ×10^-2^, proportion = 8.7%) and APOF (OR [95% CI] = 0.98 [0.96, 1.00], *p* = 4.59 ×10^-2^, proportion = 15.7%) partially mediated the causal effect between LTL and COVID-19 hospitalization. FAS (OR [95% CI] = 0.98 [0.97, 1.00], *p* = 4.56 ×10^-2^, proportion = 8.0%) and MPO (OR [95% CI] = 0.98 [0.97, 1.00], *p* = 2.13 ×10^-2^, proportion = 8.1%) partially mediated the causal effect between LTL and COVID-19 severity.

Figure created by the biorender.com

## 4 Discussion

In this systematic investigation of the role of LTL in COVID-19 phenotypes based on genetic analysis and MR, our findings suggested that LTL shortening is related to an increased risk of COVID-19. We first revealed the genetic association between LTL and COVID-19 phenotypes. Then, we clarified the significant shared genetic etiology between LTL and COVID-19 outcomes and identified many known and novel risk SNPs, which previous studies have not revealed. Then, MR analysis inferred and suggested the potential causalities from shorter LTL to a higher risk of COVID-19 phenotypes. Meanwhile, these potential causalities between LTL and COVID-19 outcomes remained significant after adjusting for possible confounders such as BMI, smoking behavior, and educational attainment. Herein, causal evidence signaled that BMI and smoking were associated with a shorter LTL and that educational level was correlated with a longer LTL. We also found that LTL partially mediates the relationship between various common risk factors (BMI, smoking, educational level) and COVID-19 outcomes. Moreover, we explored the important mediating role of plasma proteins between LTL and COVID-19 outcomes. Furthermore, a series of vital genes and biological pathways associated with LTL and COVID-19 phenotypes were identified, offering a theoretical basis for future studies on the specific mechanisms of LTL in the progression of COVID-19 pathology.

Telomeres are responsible for maintaining the integrity of the genome. They are considered to be a biomarker of temporal aging as they gradually decrease under stimuli such as oxidative stress and inflammation following birth. This age-related fluctuation in LTL has been associated with various aging-related diseases such as diabetes, hypertension, Alzheimer's disease, and cancer [7]. Thus, a reduction in LTL is considered an essential determinant of changes in one's lifespan and chronological diseases [5]. Recent observational studies have shown a strong association between LTL and COVID-19 susceptibility and severity. For example, in an observational study from the UK Biobank consortium enrolling 6775 European participants who tested positive for SARS-CoV-2, Wang et al. found that shorter leukocyte LTL was independently associated with COVID-19-related hospitalization and severity after adjusting for several identified COVID-19 risk factors, including age and smoking [58]. Besides, they also discovered that infection with SARS-CoV-2 led to a reduction in LTL. However, limited by its small sample size, this cohort study could not establish a causal link between LTL and COVID-19 risks. Of note, Retuerto et al. [12] inferred that shorter LTL was strongly correlated with the level of several inflammatory markers in patients infected with SARS-CoV-2, such as C-reactive protein (CRP), ferritin, LDH, or D-dimers, and therefore effectively reflected COVID-19 hospitalization and severity. In addition, long-term follow-up studies determined that LTL shortening is strongly associated with persistent poor pulmonary symptoms in patients recovering from COVID-19. Aviv et al. suggested that LTL shortening in circulating inflammatory cells may exacerbate the systemic inflammatory response in SARS-CoV-2 infected patients by modulating their dynamics, development, and differentiation [59]. Conversely, Jiang [14] and Huang [60] et al. did not detect a significant causality between LTL shortening and COVID-19 outcomes in two bidirectional Mendelian randomization studies. In the two MR studies, Jiang and Huang et al. yielded negative results because the GWAS data of COVID-19 outcomes were not the most recent version (fifth rounds) [61]. In contrast, those outcomes in this study were sourced from the most recent studies (seventh rounds) that included more participants and were more closely aligned to the current real-world situation. Therefore, we used the most recent GWAS data of COVID-19 outcomes and re-examined the causal relationship between LTL and COVID-19 outcomes based on multiple genetic analyses; to the best of our knowledge, this is the first study to systematically identify the shared genetic etiology and underlying mechanism between LTL and COVID-19 outcomes from multiple perspectives. Besides, we explored the mediated effects of LTL between common risk factors (smoking, BMI, and education) and COVID-19 risks. More importantly, we identified many potentially important plasma proteins mediating the link between LTL and COVID-19 outcomes, which were reported for the first time.

Over the past five decades, the increasing prevalence of obesity has become a major health concern worldwide, given that it significantly increases the risk of chronic diseases of the endocrine, circulatory, and neurological systems, eventually leading to decreased life expectancy and increased social burden [62]. Numerous researches have validated that overweight and obesity are independent risk factors for hospitalization and death related to SARS-CoV-2 infection. Sawadogo et al. described that morbidly obese patients had an increased risk of COVID-19-associated hospitalization (OR [95% CI] = 2.53 [1.67–3.84] and death (OR [95% CI] = 2.06 [1.76–3.00] [63]. In addition, a linear dose-response correlation was observed between obesity categories and the outcomes of COVID-19. Gao et al. found a linear increase in the risk of hospital admission and death in patients with severe COVID-19 and BMIs exceeding 23 kg/m^2^ and a linear increase in the risk of ICU admission across the entire BMI range [64]. Likewise, this study evident causal relationships between BMI and COVID-19 phenotypes. Moreover, epidemiological studies have similarly established a strong independent association between obesity, subcutaneous fat thickness, and LTL[65]. Bhatt et al. documented that in addition to age, aberrant fasting glucose levels were another factor contributing to significantly shorter LTL in Asian Indian women suffering from obesity and subcutaneous fat accumulation[66]. Our findings indicated that BMI was negatively correlated with LTL and partly elevated COVID-19 risk through LTL.

During the coronavirus disease pandemic, the question of smoking and its association with the risk of acute respiratory disease has regained extensive attention. There is ample evidence that tobacco impairs the immune system by governing macrophage and cytokine responses, thereby increasing the risk of respiratory tract infections in smokers. The largest study to date from the United Kingdom reported that COVID-19 patients who currently smoked had a 1.25-fold (95% CI =1.12–1.40) increased risk of death compared to never-smokers after adjusting for age and gender [67]. Mechanistic studies speculate that tobacco may promote SARS-CoV-2 entry into the host respiratory mucosa and induce active infection by upregulating angiotensin-converting enzyme 2 (ACE2) receptor expression. An observational and Mendelian randomization study using the UK Biobank cohort similarly supported a causal effect between smoking and the risk of severe COVID-19 [68]. Our finding revealed the causal effect of smoking on COVID-19 outcomes. Smoking is also a significant risk factor for diseases linked to biological aging, as reflected by alterations in telomere length. Astuti et al. concluded that smokers had shorter LTLs than nonsmokers and that current smokers had shorter LTLs than never-smokers or former smokers and noted an inverse trend between the number of cigarettes smoked and LTL [69]. Smoking also increases the risk of metabolic [70] and psychiatric disorders [71] by accelerating telomere attrition in humans. Our results indicated that smoking was negatively correlated with LTL and indirectly increased the risk of COVID-19 by mediating LTL.

It is universally accepted that educational attainment is an underlying cause of health disparities, considering that many sociological and behavioral mechanisms influence health. Countries and regions heavily impacted by the COVID-19 pandemic have faced challenges in providing consistent and uninterrupted education to students; nevertheless, the effect of educational attainment on the risk of COVID-19 remains to be elucidated. Research suggests that low educational attainment may accelerate telomere attrition through oxidative stress stemming from psychological stress, poor environments, and unhealthy behaviors. Limited observational evidence suggests a positive association between higher educational attainment and LTL [72], [73]. Needham et al. claimed that middle-aged college graduates had an average LTL of 4% longer than high school dropouts (mean age 49 years) [74]. Alexeeff et al. evinced that the difference in LTL between high school dropouts and college graduates was equivalent to approximately seven years of additional telomere aging [75]. This study discovered a negative causal effect of education on COVID-19 susceptibility, hospitalization, and severity.

LTL and COVID-19 outcomes shared significant regional genetic correlations after partitioning the whole genome into LD-independent regions in this study. Fine-mapping and colocalization analyses reveal numerous known and novel shared SNPs between LTL and COVID-19 outcomes, among which rs144204502, rs34517439, and rs56255908 have been the most important SNPs. TK1 protein is a DNA salvage pathway enzyme that regenerates thymidine for DNA synthesis and damage [55]. It was shown that TK1 regulates the replication and transcription of the SARS-CoV-2 genome by controlling the process of thymidine nucleotide biosynthesis [76]. DNAJB protein, as a subclass of the heat shock protein 40 (HSP40) family, plays molecular chaperone-like roles, including protein folding, assembly, or disassembly of protein complexes [77]. It has been shown that loss-of-function variants of DNAJB4 may lead to the accumulation of DNAJB4 client proteins, causing muscle dysfunction and degeneration, leading to early respiratory failure [78].

TWAS analysis identified a series of important genes associated with LTL and COVID-19 risk, such as *CCHCR1, HLA-C, PMF1, LRRC37A, CASTOR3, IFNAR1, SLC25A44, DR1*, and *MACF1*. Coiled-coil alpha-helical rod protein 1 (*CCHCR1*) is a psoriasis susceptibility gene and plays a decisive role in controlling mRNA metabolism by interacting with mRNA uncapping protein 4 [79]. Erola Pairo-Castineira et al. suggested that the *CCHCR1* locus is associated with life-threatening complications in patients with advanced COVID-19 [80]. Human leukocyte antigens (*HLA*), which are genes located within the major histocompatibility complex (MHC), play critical roles in immune functions, including antigen recognition and presentation [81]. Recent research indicates that variations in the HLA locus are closely linked to susceptibility to COVID-19, as well as the severity and mortality of the disease [82]. Furthermore, multiple biological pathways significantly enriched in LTL and COVID-19 outcomes were identified, including protein transmembrane transport, cellular aldehyde metabolic process, mitotic G1 DNA damage checkpoint signaling, host environment, biological process involved in interaction with host, viral entry into host cell, antigen processing and presentation of peptide antigen, regulation of phosphatase activity, and protein targeting to lysosome. These pathways regulate immune responses and maintain chromosome and telomere stability, cellular signaling, and enzymatic activity modifications. The results of this study lay a theoretical basis for future exploration of the detailed mechanisms of LTL in the pathological process of COVID-19.

Finally, six plasma proteins were found to mediate the causal effect between LTL and COVID-19 outcomes, such as BDNF, QPCT, FAS, MPO, SFTPB, and APOF. Serum Brain-Derived Neurotrophic Factor (BDNF) is the most abundant protein in the human brain and plays a crucial role in neuronal growth and synaptic plasticity. Research indicates that blood concentrations of BDNF rise following infection with COVID-19 and could act as an early biomarker for forecasting the pathogenesis of COVID-19 in adolescents [83]. Luciele Guerra Minuzzi et al. [84] showed that serum BDNF levels are useful for predicting prognostic deterioration in COVID-19, especially in male patients. Kiarash Saleki et al. [85] demonstrated that the Fas/Fas ligand pathway mediates neutrophilia and lymphopenia in COVID-19 patients and is significantly linked with pulmonary complications and increased mortality rates. MPO protein is upregulated early during COVID-19 as a critical indicator of neutrophil activity. It plays a significant role in the initial phase and progression of COVID-19 by modulating neutrophil function and facilitating the formation of neutrophil extracellular traps [86]. Liliia Fishchuk et al. [87] indicated that variants in the SFTPB gene are associated with an elevated risk of developing acute respiratory distress syndrome in COVID-19 patients.

However, this study has several limitations. First, the exposure and outcome data in this study are from European populations, limiting our findings' generalizability to other races or populations. Second, the quality of current MR analyses relies heavily on the quality and coverage of the genetic data used. Thus, the conclusions are subject to change due to incompleteness or errors in GWAS data. In addition, MR operates under the premise that genetic variations' impact on exposure remains unaffected by an individual's environmental or lifestyle variables. Nonetheless, practical observations reveal that environmental determinants can indeed alter the dynamics between genetic predispositions and disease manifestations. Last but not least, there is a small overlap in this study's exposure and outcome data (partly from the UK Biobank), which may partially impact TSMR conclusions, although we consider this potential limitation and used approaches to adjust for it.

## 5 Conclusion

In summary, this is the first study to uncover the full genetic association between LTL and COVID-19 outcomes. Mediation analysis signaled a mediating role for LTL in the causal relationship between BMI, smoking level, educational attainment, and the risk of COVID-19. In addition, the mediating effect of plasm proteins LTL and COVID-19 outcomes were explored. Furthermore, the SNPs, genes, and biological pathways identified in this paper offer a partial theoretical basis for future research to unravel the pathological mechanisms that connect LTL to COVID-19.

## Declaration

All data used by this study were publicly available from participant studies with the approval of the ethical standards committee related to human experimentation. No additional ethical approval was required in this study.

## Consent for publication

All authors consented to the submission and publication of this study.

## Author contributions

Zhixiong Liu, Peng Luo, and Quan Cheng conceived and designed the research. Jingwei Zhang and Jie Wen wrote the first draft of the manuscript. Jingwei Zhang, Jie Wen, Ziyu Dai, Hao Zhang, Nan Zhang, Ruoyan Lei, Zhixiong Liu, Peng Luo, and Quan Cheng contributed to data acquisition, data analysis, and interpretation. Jingwei Zhang, Jie Wen, Ziyu Dai, Hao Zhang, Nan Zhang, Ruoyan Lei, Zhixiong Liu, Peng Luo, and Quan Cheng contributed to the revision of the paper. All authors contributed to the article and approved the final manuscript.

## Funding

This research was funded by the Hunan Youth Science and Technology Talent Project (NO.2023RC3074) and the 10.13039/501100004735Natural Science Foundation of Hunan Province (NO. 2023JJ30971).

## CRediT authorship contribution statement

**Jingwei Zhang:** Conceptualization, Data curation, Formal analysis, Funding acquisition, Investigation, Methodology, Project administration, Writing – original draft. **Jie Wen:** Conceptualization, Data curation, Formal analysis, Investigation, Methodology, Project administration, Writing – original draft. **Ziyu Dai:** Data curation, Formal analysis, Project administration, Resources, Software. **Nan Zhang:** Investigation, Project administration, Writing – original draft. **Hao Zhang:** Resources, Software, Writing – original draft. **Ruoyan Lei:** Data curation, Formal analysis, Resources, Software. **Zhixiong Liu:** Conceptualization, Methodology, Resources, Software, Supervision, Validation, Visualization. **Quan Cheng:** Conceptualization, Formal analysis, Funding acquisition, Software, Supervision, Validation, Visualization. **Peng Luo:** Conceptualization, Data curation, Formal analysis, Resources, Software, Supervision, Visualization.

## Declaration of Competing Interest

None.

## Data Availability

All data used in this study were available in the original research. Data generated in this study were included in the main text and supplementary files.

## Appendix A Supplementary material

Supplementary material.Fig. 1. TWAS identifies the important genes between LTL and COVID-19 outcomes. (A) TWAS of shared genes associated with LTL and all COVID-19 outcomes in the reverse direction. (B) Protein-protein interaction (PPI) plot for shared hub genes and predicted genes. Light blue color represents COVID-19 susceptibility; Orange color represents COVID-19 hospitalization; Pink color represents COVID-19 severity.

.

Supplementary material.Fig. 2. TWAS identifies the potential biological pathways between LTL and COVID-19 susceptibility (A), hospitalization (B), and severity (C).

.

Supplementary material.Fig. 3. Leave-one-out analysis determines the reliability of the MR results.
