# Supplementary figures and images for: Causal association and shared genetics between telomere length and COVID-19 outcomes: New evidence from the latest large-scale summary statistics
Source: Comput Struct Biotechnol J. 2024 May 10;23:2429–41. doi: 10.1016/j.csbj.2024.05.012 (PMC11176559; doi:10.1016/j.csbj.2024.05.012)

**(A)****Susceptibility****Hospitalization****Severity**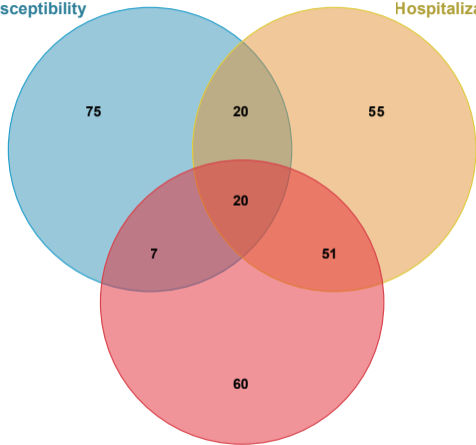**(B)**

Co-expression

Genetic interaction

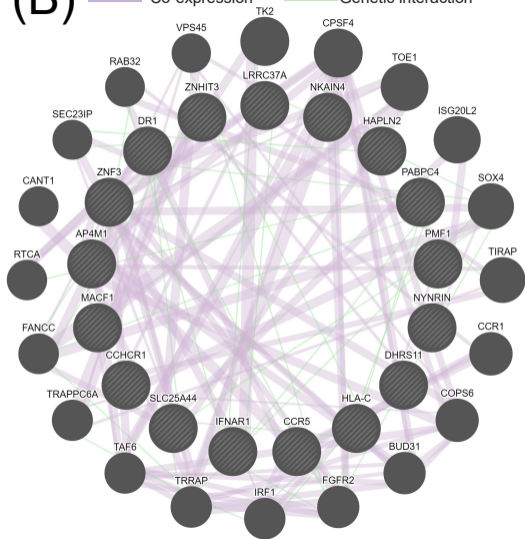

Supplement: Supplementary file 2 — Supplementary material.Fig. 2. TWAS identifies the potential biological pathways between LTL and COVID-19 susceptibility (A), hospitalization (B), and severity (C). [file mmc2.pdf]

(A) Susceptibility

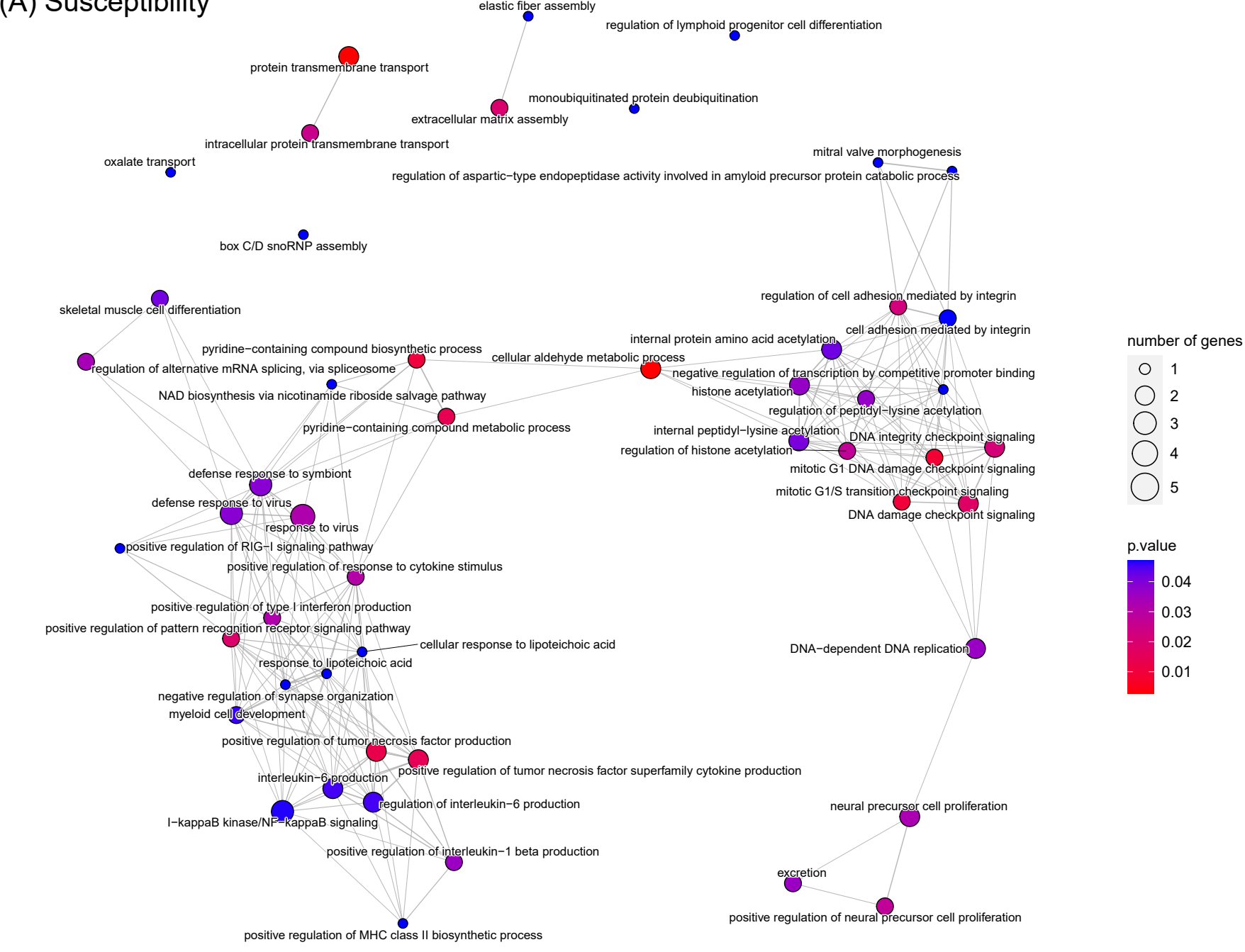

(B) Hospitalization

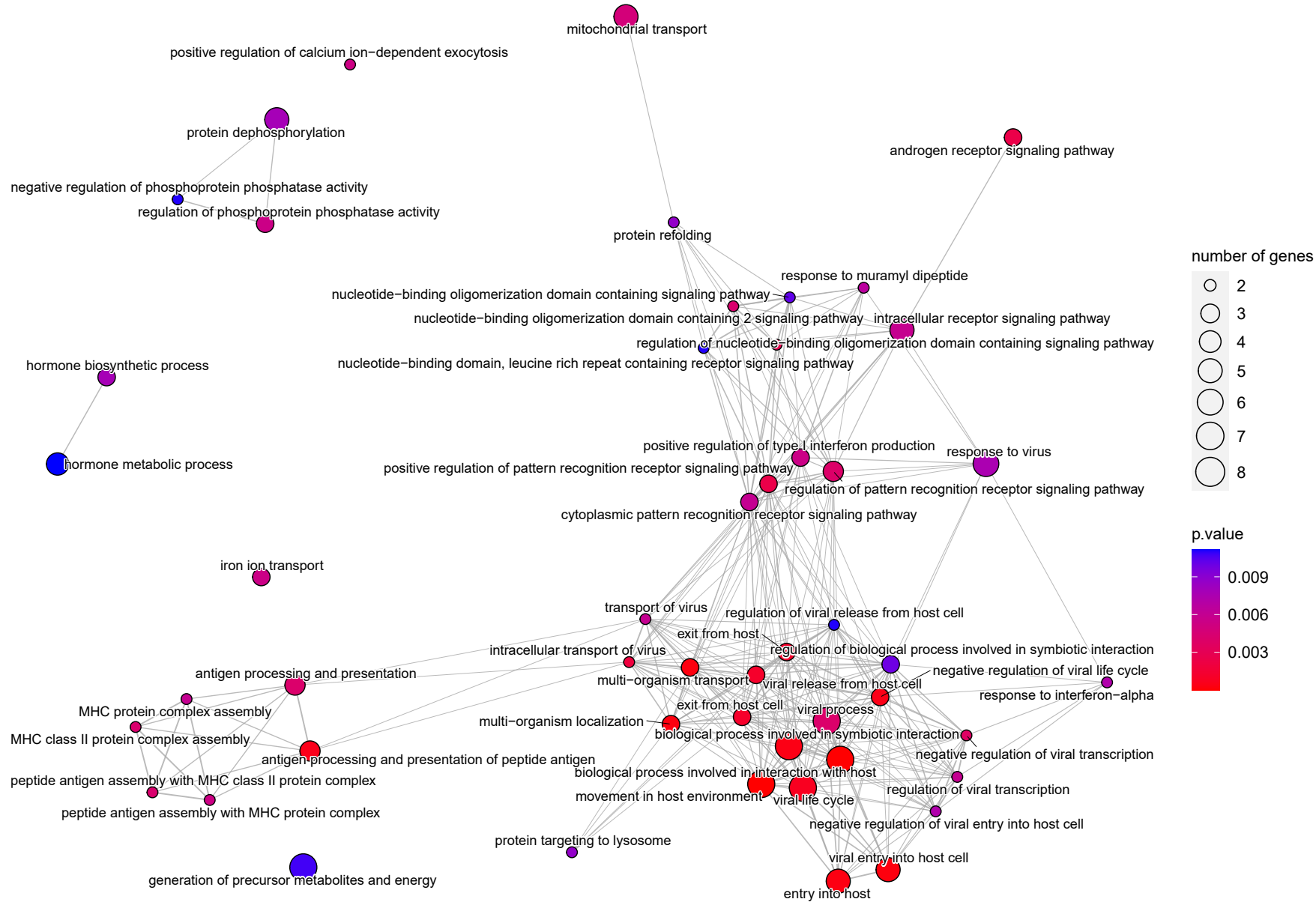

(C) Severity

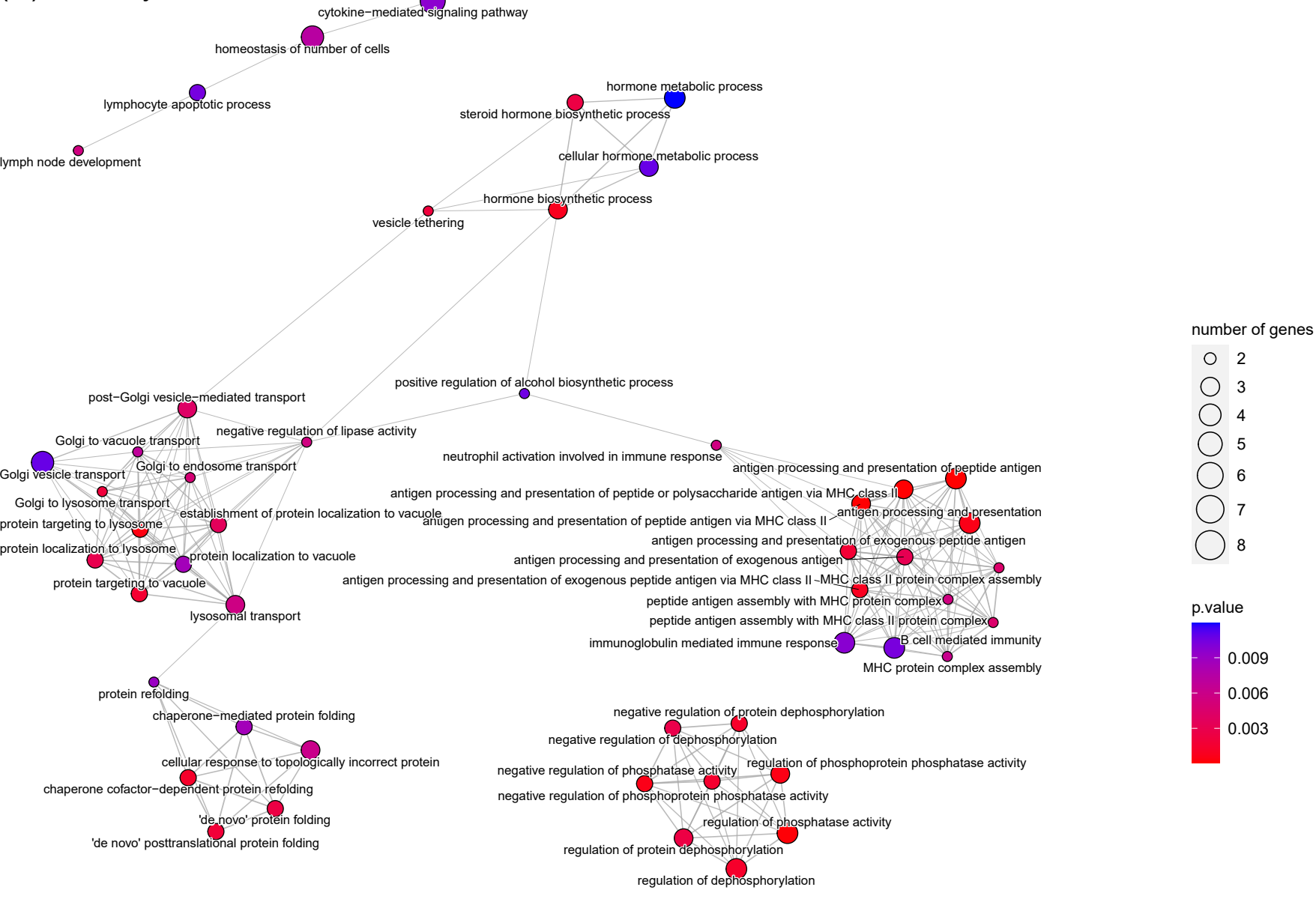

Supplement: Supplementary file 3 — Supplementary material.Fig. 3. Leave-one-out analysis determines the reliability of the MR results. [file mmc3.pdf]

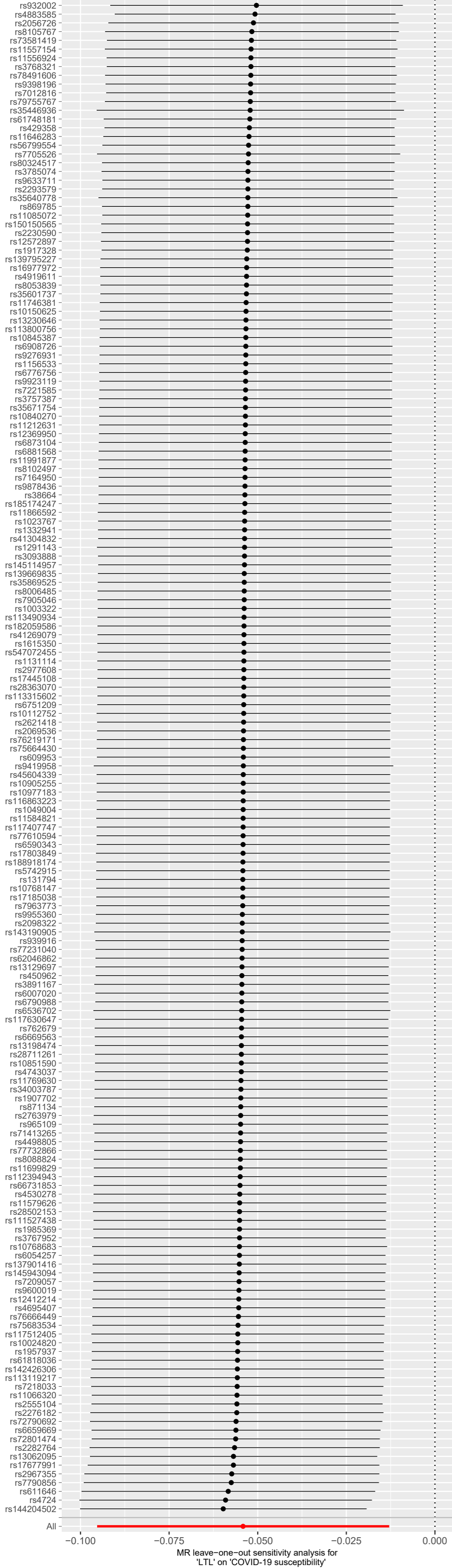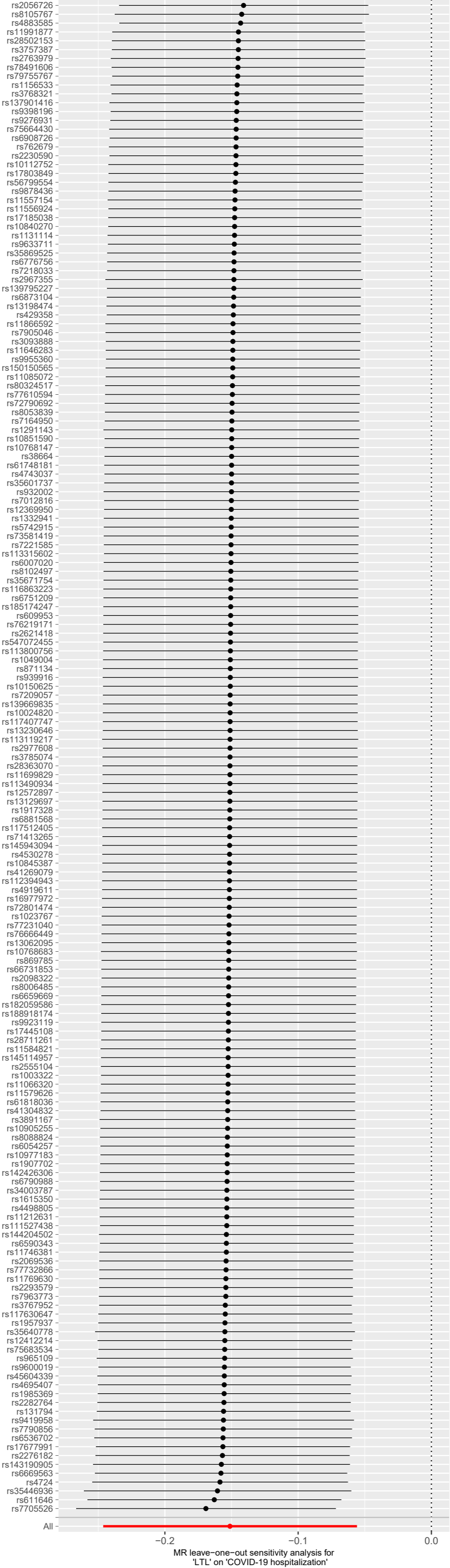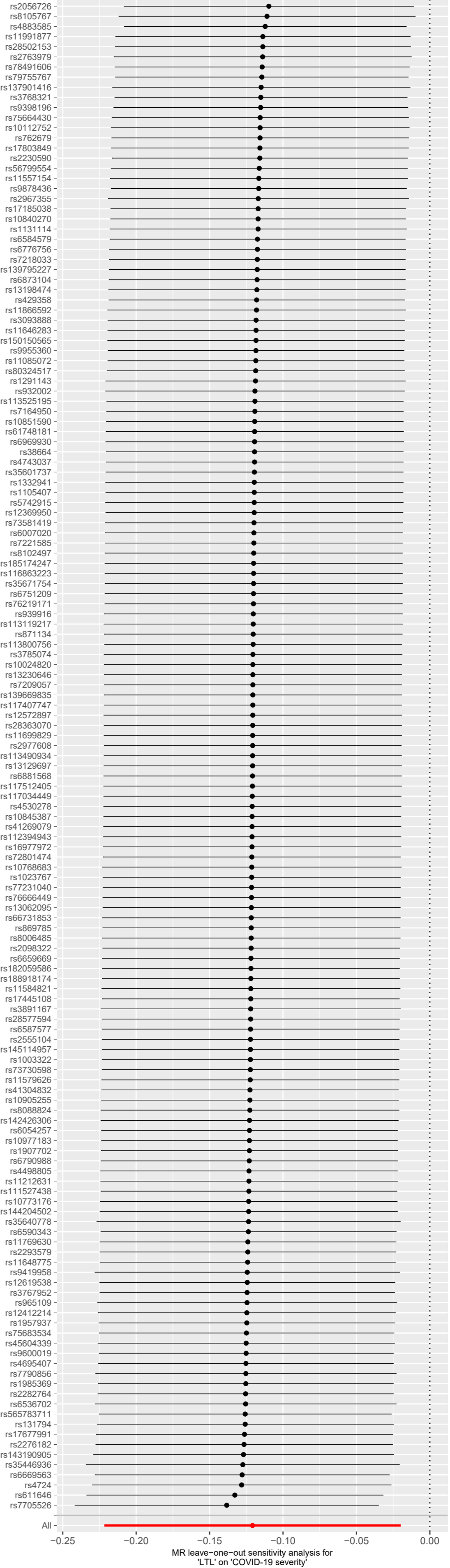

Supplement: Supplementary file 4 — Supplementary material [file mmc4.pdf]
